# Point of view and expectation of parents with children diagnosed with autism spectrum disorder enrolled in regular and special schools

**DOI:** 10.1192/j.eurpsy.2021.544

**Published:** 2021-08-13

**Authors:** B. Ferreira, F. Fernandes

**Affiliations:** School Of Medicine, universidade de São Paulo, São Paulo, Brazil

**Keywords:** Autism, autism in School, Job Market for autistic individual, Covid-19 pandemic and autism

## Abstract

**Introduction:**

Autism Spectrum Disorder (ASD) is a neurodevelopmental disorder that can cause social and professional harm in an individual. Many teachers are unprepared to receive students with ASD. They find it difficult to communicate with other professionals or to comfort the family. And parents often do not feel that their children at school benefit socially and in their academic skills.

**Objectives:**

To know the point of view and expectations of parents with children with Autism Spectrum Disorder about the academic future and professional life.

**Methods:**

An online questionnaire was applied with 38 multiple choice questions about the future academic perspectives and expectations of their children, the job market and about school adaptations and the school year during the COVID-19 pandemic.

**Results:**

So far, 16 parents of a Speech-Language Pathology laboratory in which they assist children with Autism Spectrum Disorder (ASD) have answered the questionnaire. When asked if there were teachers who work with TEA individuals, nine guardians 56.3% answered that they did not, 50% of the guardians said they had received adapted material during the COVID-19 pandemic, 100% of the guardians believe that their child will finish high school, 93.8% of those in charge do not believe that the job market is prepared for individuals with ASD.

**Conclusions:**

Parents are dissatisfied with the education their children receive and most of them did not get adequate support during the COVID-19 pandemic. There are also low expectations for the future in the job market.

